# Chemokines in Physiological and Pathological Bone Remodeling

**DOI:** 10.3389/fimmu.2019.02182

**Published:** 2019-09-13

**Authors:** Laura J. Brylka, Thorsten Schinke

**Affiliations:** Department of Osteology and Biomechanics, University Medical Center Hamburg-Eppendorf, Hamburg, Germany

**Keywords:** bone remodeling, chemokines, osteoblasts, osteoclasts, osteoimmunology

## Abstract

The bone matrix is constantly remodeled by bone-resorbing osteoclasts and bone-forming osteoblasts. These two cell types are fundamentally different in terms of progenitor cells, mode of action and regulation by specific molecules, acting either systemically or locally. Importantly, there is increasing evidence for an impact of cell types or molecules of the adaptive and innate immune system on bone remodeling. Understanding these influences is the major goal of a novel research area termed osteoimmunology, which is of key relevance in the context of inflammation-induced bone loss, skeletal metastases, and diseases of impaired bone remodeling, such as osteoporosis. This review article aims at summarizing the current knowledge on one particular aspect of osteoimmunology, namely the impact of chemokines on skeletal cells in order to regulate bone remodeling under physiological and pathological conditions. Chemokines have key roles in the adaptive immune system by controlling migration, localization, and function of immune cells during inflammation. The vast majority of chemokines are divided into two subgroups based on the pattern of cysteine residues. More specifically, there are 27 known C-C-chemokines, binding to 10 different C-C receptors, and 17 known C-X-C-chemokines binding to seven different C-X-C receptors. Three additional chemokines do not fall into this category, and only one of them, i.e., CX3CL1, has been shown to influence bone remodeling cell types. There is a large amount of published studies demonstrating specific effects of certain chemokines on differentiation and function of osteoclasts and/or osteoblasts. Chemokine signaling by skeletal cells or by other cells of the bone marrow niche regulates bone formation and resorption through autocrine and paracrine mechanisms. *In vivo* evidence from mouse deficiency models strongly supports the role of certain chemokine signaling pathways in bone remodeling. We will summarize these data in the present review with a special focus on the most established subsets of chemokines. In combination with the other review articles of this issue, the knowledge presented here confirms that there is a physiologically relevant crosstalk between the innate immune system and bone remodeling cell types, whose molecular understanding is of high clinical relevance.

## Introduction

### Skeletal Development and Remodeling

The skeleton consists of more than 200 differently shaped elements, which form by two distinct types of ossification. More specifically, whereas intramembranous ossification, involving direct differentiation of mesenchymal stromal cells into bone-forming osteoblasts, occurs primarily in the skull, most skeletal elements develop by endochondral ossification, where a cartilage intermediate is formed first (1). Here the mesenchymal cells condensate to form chondrocytes, which further differentiate into a hypertrophic state to produce a mineralized cartilage matrix. This initial step occurs in the center of the developing bones, and the subsequent replacement of cartilage by bone generates two zones, i.e., the growth plates, where chondrocytes continue to undergo a specific differentiation program from both sides toward the center (2). This program generates, similar to the initial step, hypertrophic chondrocytes producing mineralized cartilage, which is remodeled into bone by osteoclasts and osteoblasts. Importantly, this transition requires vascularization of these areas to allow invasion of the two cell types (3). Not only during skeletal development and growth, but also thereafter, there is a continuous remodeling of the bone matrix, which takes place throughout adult life (4). This steady renewal process, which is required to maintain skeletal integrity over decades, is mediated by two antagonistically acting cell types, i.e., osteoblasts and osteoclasts, which are fundamentally different in terms of progenitor cells, morphology, mode of action and regulatory molecules affecting their differentiation and function.

More specifically, osteoclasts represent a unique cell type with the ability to resorb mineralized matrix. Osteoclasts are generated by fusion of hematopoietic progenitors of the monocyte/macrophage lineage, which results in huge multinucleated cells and ensures, after attachment to mineralized bone, the formation of a large ruffled surface being required for proper resorption (5). The function of osteoclasts depends on two major mechanisms, i.e., extracellular acidification and secretion of matrix-degrading enzymes. Their dysfunction causes osteoclast-rich osteopetrosis, a severe disorder of early childhood, which requires immediate treatment (6). More specifically, the respective patients are strongly affected by impaired hematopoiesis and immunity, since their bone marrow is replaced by non-resorbed bone and marrow fibrosis. Importantly, if caused by an intrinsic osteoclast defect, which applies for the majority of cases, osteopetrosis is curable by hematopoietic stem cell (HSC) transfer. Besides osteoclast-rich osteopetrosis, there are additional patients, where osteoclasts are not generated. This specific disorder, i.e., osteoclast-poor osteopetrosis, can be caused by inactivation of genes encoding either the transmembrane protein receptor activator of nuclear factor κB (RANK) or RANK ligand (RANKL) (7). Confirmed by a huge number of *in vitro* and *in vivo* studies it is well-established that binding of RANKL, which is primarily expressed by osteoblast lineage cells, to RANK expressed by osteoclast progenitor cells is the most relevant trigger for osteoclast differentiation and bone resorption (8). Most importantly, *in vitro* formation of bone-resorbing osteoclasts does not occur in the absence of RANKL, and mice deficient for RANKL display severe osteopetrosis as they do not develop osteoclasts (9, 10). Moreover, the molecular interaction between RANK and RANKL can be physiologically counteracted by osteoprotegerin (OPG), a soluble protein acting as a decoy receptor of RANKL.

As stated above, osteoblast lineage cells are fundamentally different from osteoclasts and are physiologically regulated by other sets of molecules. Osteoblasts derive from mesenchymal progenitors residing in the bone marrow. They accumulate in larger groups of cells to simultaneously produce the extracellular matrix of bone, which is initially unmineralized. This matrix, termed osteoid, primarily consists of type I collagen, but also contains several additional proteins, such as serum-derived fetuin-A or locally produced matrix proteins, some of them selectively expressed by osteoblasts (11). During the process of matrix mineralization, which is still not fully understood at the molecular level, a subset of osteoblasts is embedded into the mineralized bone matrix to terminally differentiate into osteocytes (12). This third bone cell type is again unique in its morphology, since it forms long cytoplasmic extensions, which are connected to other osteocytes, but also to the bone surface. Osteocytes are known to regulate skeletal remodeling, for instance by producing sclerostin, a physiologically relevant inhibitor of osteoblast activity, whose mutational inactivation causes osteosclerosis, i.e., high bone mass due to excessive bone formation (13). The anti-osteoanabolic activity of sclerostin is molecularly explained by interaction with the transmembrane protein LRP5 (Low-density lipoprotein receptor-related protein 5), which physiologically promotes bone formation (14, 15). Although there are many other systemic or local regulators of bone formation known to date, it is evident that osteoclasts and osteoblasts have to be regarded separately when it comes to influences of specific molecules. Importantly however, there is hallmark evidence for a molecular communication between the two bone remodeling cell types, which is mediated by the RANKL/OPG system, but also by osteoanabolic molecules derived from osteoclasts (16).

The most prevalent bone remodeling disorder, i.e., osteoporosis, is characterized by systemic bone loss causing increased risk of skeletal fractures. Although there are various causes for osteoporosis in different patient groups, the disease is generally explained by a relative increase of bone resorption over bone formation. Given the differential regulation of osteoclasts and osteoblasts described above, there are two distinct options to treat osteoporosis, either inhibiting osteoclast differentiation and/or activity by anti-resorptives (RANKL neutralization or bisphosphonates) or stimulating osteoblast-mediated bone formation by osteoanabolic medication (teriparatide or sclerostin neutralization). With respect to osteoporosis management, it is also important to state that prolonged anti-resorptive treatment by interfering with physiological remodeling and renewal of the bone matrix may have adverse effects on skeletal integrity, i.e., increased fracture risk despite high bone mass. Therefore, osteoanabolic treatment options or their combination with anti-resorptives might be the preferable strategy for osteoporotic patients in the future (17). On the other hand, there are specific pathologies, where excessive osteoclastogenesis is the primary clinical problem, which are most effectively treated by either bisphosphonates or antibody-mediated blockade of RANKL. These include multiple myeloma (MM), various skeletal metastases, but also different inflammatory disorders, as discussed below (18).

### Molecular Crosstalk Between Bone and the Immune Cells

An interaction between bone remodeling and the immune system is supported by several arguments. First, as discussed above, osteoclasts derive from hematopoietic progenitor cells and therefore represent a highly specialized immune cell. Second, the progenitors of both, osteoclasts and osteoblasts are located in the bone marrow, where they are in direct contact with progenitor or memory cells of the immune system. Third, the major pro-osteoclastogenic cytokine RANKL is not only expressed by osteoblast lineage cells, but also by activated T cells and B cells, and it not only promotes osteoclast differentiation, but also influences different immune cell types (19–21). Fourth, there are various reports showing that bone remodeling cell types affect immune cell differentiation, whereas many different cell populations of the innate and adaptive immune system were found to affect bone remodeling (22). Finally, there are several inflammatory disorders with a negative influence on bone mass, most of them associated with excessive bone resorption (23). Understanding the respective interactions at a molecular level is the focus of an emerging research area known as osteoimmunology, which has led to the discovery of specific cytokines with a remarkable influence of bone remodeling (24).

For example, there is hallmark evidence for a strong positive impact on osteoclastogenesis mediated by TNF-α, IL-1, IL-6, or IL-17. On the other hand, some cytokines were found to have an opposite effect, one of them IL-33, which inhibits osteoclast differentiation *in vitro* and *in vivo* (25, 26). It is important to state however, that there is a high complexity behind these influences, i.e., there are many conflicting results reported in the literature (22). Since this is potentially explained by different experimental settings and/or co-administration of other cytokines, these collective findings essentially suggest that the influence of inflammatory cytokines on bone remodeling cell types strongly depends on their maturation stage and the presence or absence of co-stimulatory signals. It is therefore even more important to refer to clinical data highlighting the specific role of certain cytokines in the context of osteoimmunology. For instance, the severe bone affection in patients with mutations of *IL1RN*, encoding an IL-1 receptor antagonist, essentially confirms the human relevance of IL-1 actions on skeletal cell types (27). Moreover, there is one particular cytokine, i.e., IL-17, where accumulating evidence over the last years strongly suggests a key role in the pathogenesis of bone loss in various inflammatory disorders. These include rheumatoid and psoriatic arthritis, periodontitis, inflammatory bowel disease and primary sclerosing cholangitis (28–32). At a molecular level, IL-17, primarily produced by Th17 cells, has been shown to promote osteoclastogenesis indirectly by inducing RANKL production in synovial fibroblasts or osteoblasts.

Since this cumulative knowledge has been summarized in various comprehensive review articles, the focus of the present article is solely related to another group of immune cells regulators, i.e., chemokines. More specifically, we will discuss the current knowledge regarding the impact of specific chemokines and their receptors on skeletal cell types. This includes direct or indirect influences on osteoclastogenesis and bone resorption, effects on osteoblast lineage cells and endochondral ossification. Moreover, since these interactions may be more relevant in the context of specific pathologies, we will further focus on the impact of chemokines on inflammatory bone loss, behavior of metastatic tumor cells and cancer-induced osteolytic lesions. In fact, certain cancers, such as breast, lung and prostate cancers, home predominantly to the bone marrow niche (33). Here the disseminated cancer cells can undergo dormancy and stay quiescent for up to several years until they start to proliferate again, colonize the bone marrow niche and form metastases (34). These bone metastases often cause osteolytic lesions by inducing osteoclasts to resorb bone. The underlying mechanisms of bone homing, dormancy and exit from dormancy, as well as osteolysis are not yet fully understood. There is however strong evidence showing that specific chemokines are involved in the homing of metastatic cancer cells to the bone marrow and also in osteolysis. Likewise, chemokines have also been shown to be involved in osteolytic bone destruction occurring in multiple myeloma, a type of cancer caused by uncontrolled proliferation of plasma cells in the bone marrow (35).

### Chemokines as Key Regulators of the Innate Immune System

Chemokines are homologous heparin-binding molecules with a molecular mass of 8–12 kDa, which are involved in many biological processes, including homing of immune cells, development, inflammation and angiogenesis (36–39). Almost 50 chemokine ligands are known, which are classified into four subfamilies according to their structure. The chemokine nomenclature refers to the first two highly conserved cysteine residues. The largest family is comprised by the C-C-chemokines in which the two cysteines are adjacent. The second largest group is represented by the C-X-C-chemokines, in which the cysteines are separated by one amino acid. CX3CL1/fractalkine, the only member of the C-X3-C family, contains three amino acids between the cysteines, whereas the two chemokines of the X-C family only have one cysteine. The nomenclature of the corresponding receptors is according to their chemokine ligands (however, note that CX3CL1 also binds CCL26). There are 19 classical chemokine receptors known, which are all G-protein-coupled receptors (GPRCs) containing a rhodopsin-like 7-transmembrane domain structure. The interactome between chemokines and their receptors is quite complex, due to receptor/ligand promiscuity and redundancy. Several different chemokines can bind to the same receptor, and some chemokines are able to bind to more than one receptor. Furthermore, chemokines can form homo- and heterodimers or oligomers, which can lead to different signaling responses compared to the monomer (36). Another level of complexity is added by atypical chemokine receptors (ACKR), also known as chemokine decoy receptors. There are four atypical chemokine receptors (ACKR) known (ACKR1-ACKR4) (40–42). These receptors do not induce classical G-protein coupled signaling, but internalize the ligand and either induce ligand degradation, or transport the ligand to the other side of the cell. Similar to canonical chemokine receptors, ACKRs can dimerize and oligomerize with other chemokine receptors, and in this manner modulate chemokine signaling (42). Intriguingly, the central regulatory mechanism in osteoimmunology, i.e., RANKL/RANK signaling is also controlled by a decoy receptor, OPG.

Functionally, chemokines are known to form chemotactic gradients (with the exception of membrane-bound CX3CL1 and CXCL16) in order to guide cells toward the highest chemokine concentration (43). In this manner, they orchestrate cell migration in various biological processes. Chemokines can have major physiological functions, such as the well-known CXCL12/CXCR4 axis, which is crucial for homing of HSC in the bone marrow niche (44, 45). However, chemokines are mostly known for their regulatory functions of the immune system during inflammation, where they play important roles for the innate as well as the adaptive immune system (46, 47).

Importantly, the CXC-family of chemokines can be subdivided into two groups, depending on the presence of a specific motif which has functional implications. CXC-chemokines carrying a glutamate-arginine-leucine (ELR) motif near the N-terminus, are all agonists for the receptors CXCR1 and CXCR2, which can both be found on neutrophils (46, 48). Therefore, ELR-positive chemokines are crucial for neutrophil recruitment during wound repair or bacterial defense. Additionally, the presence of the ELR motif also determines their role in angiogenesis. Generally, chemokines containing the ELR motif are angiogenic, whereas ELR-negative chemokines are angiostatic, with the exception of CXCL12, which is an ELR-positive angiogenic chemokine (49). As most literature on chemokine function in angiogenesis focuses on the role of the CXC-chemokine family, CXC-chemokines are regarded as “the regulatory link between inflammation and angiogenesis” (50–53). However, CC-chemokines were shown to also regulate angiogenesis. For instance the pro-angiogenic chemokine CCL2 activates CCR2 on endothelial cells (54).

## Influence of CC-chemokines on Bone Remodeling in Health and Disease

**Figure 1:**
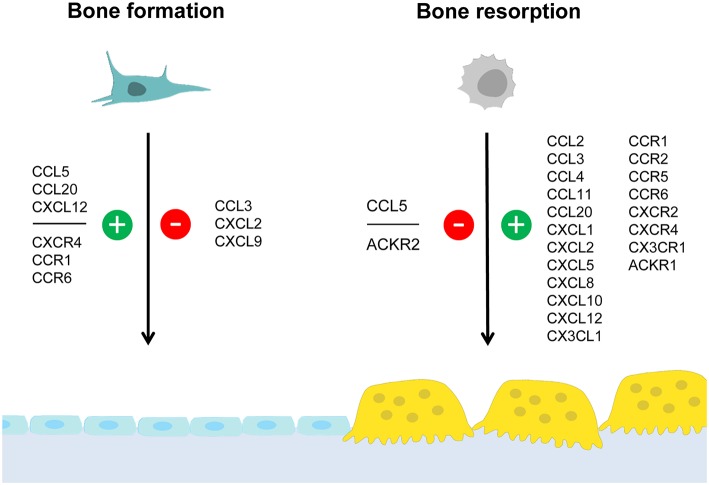
Summary of chemokine influences on bone formation and/or resorption. Osteoblasts and osteoclasts are distinctive cell types required for bone formation and bone resorption, respectively. Whereas osteoblasts (left) derive from mesenchymal stem cells, osteoclasts (right) are generated by fusion of hematopoietic progenitor cells. This simplified schematic representation summarizes chemokines and chemokine receptors for which an influence on either bone formation and/or bone resorption was established. Positive influences are indicated in green (with the “+” symbol) whereas negative influences are indicated in red (with the “–” symbol). The data supporting these influences, the underlying mechanisms and the impact on pathological conditions are discussed in the text.

There are several chemokines of the CC-family, which were shown to influence skeletal remodeling in physiological and pathological conditions. The most established ones are CCL2, CCL3, and CCL20, which will be discussed separately below. Based on numerous publications from different investigators it is evident that these chemokines share the ability to promote osteoclastogenesis, which is supported by cell culture studies, analysis of mouse deficiency models and, to some extent, by patient analyses. On the other hand, our own comparative analysis of *Ccl2*- and *Ccl5*-deficient mice revealed that these two chemokines have different influences on skeletal remodeling cell types. Since it was further remarkable that the osteoblast-related phenotype of *Ccl5*-deficient mice diminished with age, we will discuss these findings as an example of functional redundancy. Moreover, although the complexities of specific chemokine influences on either osteoclast or osteoblast differentiation are discussed in the following sections, we have summarized the current knowledge in a simplified schematic representation (Figure 1).

### CCL2

The pro-inflammatory chemokine CCL2 (also known as MCP-1), attracts dendritic cells, memory T cells and basophils via its receptor CCR2 (46). CCL2 plays a crucial role in bone remodeling, as demonstrated by studies involving mice deficient for *Ccl2* or *Ccr2* (55–57). Both mouse models show an increased bone mass due to decreased bone resorption, lower osteoclast numbers and a defect in osteoclast formation and function. *Ccl2*^−/−^ mice have a milder phenotype compared to *Ccr2*^−/−^ mice, which is probably due to the fact that CCR2 binds multiple ligands (55–57). The skeletal phenotype of mice deficient for *Ccr2* was shown to be solely caused by a decrease in bone resorption, as osteoblasts in these mice were not affected. The activation of CCR2 signaling in osteoclast progenitor cells was shown to stimulate NF-κB and ERK1/2 signaling, thereby increasing the expression of RANK and making the cells more susceptible to differentiate into mature osteoclasts (55). In line with this, it was shown that osteoclast progenitor cells from *Ccl2*-deficient mice exhibited a decreased expression of RANK and a decreased sensitivity toward stimulation with RANKL (56).

To further investigate the role of CCR2 signaling in bone, *Ccr2*^−/−^ mice were subjected to ovariectomy (OVX). In wildtype mice, CCR2 expression was increased in osteoclast progenitor cells. Mice deficient for *Ccr2* were resistant to bone loss after OVX, suggesting a role for CCR2 signaling in estrogen-deficiency mediated osteoporosis. As both, *Ccr2*^−/−^ and wildtype OVX mice, showed similar numbers of bone-marrow pre-osteoclasts, the recruitment of these cells was independent of CCR2. However, as *Ccr2*^−/−^ OVX mice showed decreased bone marrow RANK expression compared to wildtype OVX mice, CCR2 plays a role in osteoclast formation in the bone marrow. Also, in *Ccr2*^−/−^ OVX mice only CCL2 serum levels were elevated, but not those of other chemokines were altered. Thus, the reduction in bone resorption in *Ccr2*^−/−^ OVX mice was mainly caused by a lack of CCL2/CCR2 signaling. Taken together, the enhanced differentiation of preosteoclasts to osteoclasts due to increased CCR2 expression and the hereby-resulting increased RANK expression induced systemic bone loss after ovariectomy. This finding might be clinically relevant, as *Ccl2* was shown to be among the most strongly induced genes in human osteoporotic bone (58). One way to treat osteoporosis is by injection of the bone anabolic peptide parathyroid hormone (PTH). PTH stimulates bone formation, but also induces bone resorption by osteoclasts through stimulation of M-CSF and RANKL expression. Interestingly, *Ccl2* was shown to be the most strongly induced gene in osteoblasts upon PTH treatment in rats (59). When *Ccl2*-deficient mice were treated with PTH, both the anabolic effect as well as the increase in osteoclast number were reduced, indicating that the anabolic effect depends on stimulation of osteoclast progenitor cells with both RANKL and CCL2 (59–61).

CCL2 was also shown to be involved in other pathological conditions. Osteoblastic CCL2 induced the migration of CCR2-expressing cancer cells and in this manner contributed to bone metastasis formation (62–64). Also cancer cells were reported to express CCL2, thereby increasing tumor growth and osteolysis (65, 66). Furthermore, CCL2 was shown, amongst other chemokines, to be a chemoattractant for MM cells and its expression levels in patients correlated with the occurrence of multiple bone lesions (67). Moreover, inflammatory mediators or bacteria were found to induce the expression of CCL2 by osteoblasts *in vitro* (68, 69) and *in vivo* (70, 71) and in this manner contribute to inflammatory bone loss.

A physiological role for CCL2 has also been suggested in the recruitment of osteoclast precursor cells during tooth eruption (72, 73). Moreover, the expression of CCL2 was shown to be induced in osteoblasts during bone repair in a rat model of ulnar stress fracture (74). In line with its role in osteoclast differentiation, fracture healing was delayed in *Ccr2*-deficient mice, as shown by decreased numbers of infiltrating macrophages at the fracture site combined with a defect in osteoclast function (75).

Taken together, these collective data strongly suggest that CCL2, at least in mice, is involved in promoting osteoclastogenesis and bone resorption by stimulating RANK expression in a CCR2-dependent manner. Although it is worthwhile to mention, that the high bone mass and decreased osteoclastogenesis phenotype of *Ccl2*-deficient mice has been reported in three independent studies (55–57), the impact of the CCL2/CCR2 axis for human bone remodeling, osteoporosis, cancer metastases, and/or osteolytic bone destruction remains to be established.

### CCL3

A role in bone resorption has also been suggested for CCL3 (also known as MIP-1α). CCL3 binds to the receptors CCR1 and CCR5 on lymphocytes, monocytes, macrophages, eosinophils, natural killer cells and dendritic cells and was originally isolated from macrophages, but is also expressed by active osteoblasts (76). Similar to CCL2, CCL3 induces osteoclast formation in a RANK/RANKL-dependent manner, as the injection of recombinant CCL3 increased osteoclast numbers in calvariae of wildtype, but not in *Tnfrsf11a*-deficient (RANK) mice (77). Furthermore, *in vitro* experiments showed that CCL3 stimulated osteoclastogenesis directly, and indirectly by inducing RANKL expression in stromal cells and osteoblasts (78–80). Moreover, CCR1, which binds CCL3 and several other chemokine ligands, was found to be induced by RANKL in bone marrow and in RAW264.7 cells during *in vitro* osteoclast differentiation (81, 82), while treatment with the CCR1-specific antagonist MLN3897 inhibited *in vitro* osteoclastogenesis (83). CCR1 and its alternative ligand CCL9 were further reported to be the major chemokine receptor and ligand expressed in RANKL-stimulated mouse osteoclasts (84).

Similar to CCL2, CCL3 was shown to be involved in fracture healing (85). In a mouse model of femur fracture, *Ccl3* expression was increased at fracture sites, while neutralization of CCL3 delayed macrophage recruitment and fracture healing. There is also clinical evidence for a role of CCL3 in human bone remodeling. In line with its role in osteoclast differentiation, CCL3 expression in circulating monocytes correlated with low bone mineral density in patients (86). Furthermore, a cross-sectional study showed that postmenopausal osteoporotic women had elevated CCL3 serum levels compared to non-osteoporotic controls (87). CCL3 also plays a role in inflammatory bone loss, in particular in animal models of rheumatoid arthritis (RA). In an RA rat model, CCL3 expressed by macrophages recruited osteoclast progenitor cells to the distal tibia, leading to local bone destruction (88). In line with this, treatment with an anti-CCL3-antibody led to decreased disease severity in a mouse model of collagen-induced arthritis (89). Furthermore, one publication showed that B cell-derived CCL3 inhibits bone formation in RA (90). The authors demonstrated in two different RA mouse models, that B cells accumulated in subchondral bone and in the endosteal niche adjacent to osteoblasts and expressed CCL3 and other factors, which inhibited osteoblast function, while depletion of mature B cells attenuated bone loss in these mice. The authors confirmed the clinical significance of their finding by demonstrating that B cells from RA patients expressed increased levels of CCL3 and inhibited *in vitro* osteoblast differentiation.

Finally, CCL3 appears to play a major role in MM osteolysis. First of all, there is a direct causative link between MM and CCL3 expression. Malignant plasma cells overexpressing FGFR3 or with activating RAS mutation were shown to express increased levels of CCL3, as CCL3 is a downstream target of FGFR3 which signals through the RAS-MAPK pathway (91). Other studies identified CCL3 as an osteoclastogenic factor involved in the formation of osteolytic lesions in MM patients which directly affect migration and survival of MM cells (92, 93). In line with the role of the CCL3/CCR1 axis in osteoblastogenesis, CCL3 from MM cells was shown to inhibit osteoblast function, leading to uncoupling of bone formation and bone resorption (83, 94). Likewise, treatment of a humanized MM mouse model with the CCR1-specific inhibitor MLN3897, led to increased osteoblast function, decreased osteoclast formation, as well as reduced tumor burden (83). Similar studies of MM mouse models showed that the CCR1 antagonist CCX721 could decrease osteoclastic activity, osteolytic lesions and tumor formation (95). Moreover, administration of an anti-CCL3 antibody could reduce tumor growth and osteolysis (77).

In the context of the putative function of CCL3 in bone remodeling, it is further relevant to state that a remarkable bone remodeling phenotype was reported for *Ccr1*-deficient mice (96). In contrast to *Ccr2*^−/−^ mice, which display increased bone mass due to impaired osteoclastogenesis, *Ccr1*^−/−^ mice are characterized by low-turnover-osteopenia, i.e., decreased trabecular bone mass with low numbers of both, osteoclasts and osteoblasts. Furthermore, the *ex vivo* differentiation into the two cell types was impaired in *Ccr1*^−/−^ cultured bone marrow cells, indicating that chemokine signaling through CCR1 affects both arms of bone remodeling. Importantly however, the authors provided additional evidence suggesting that CCL3, even though it is a major ligand of CCR1, was not involved in the development of this phenotype. More specifically, treatment of bone marrow cells with a neutralizing anti-CCL3 antibody did not affect osteogenic differentiation, in contrast to antibodies against other ligands, including CCL5 and CCL9. Therefore, although it remains to be established, which CCR1 ligands are involved in the bone-anabolic function of CCR1, it appears that CCL3 does not induce osteoblast differentiation, but rather inhibits it, as discussed above.

Collectively, there is strong evidence for a critical impact of the CCL3 on bone remodeling cell types. In contrast to CCL2, CCL3 does not only promote osteoclastogenesis, but also has a negative influence on bone formation by osteoblasts. Especially in the context of MM, where CCL3 expression might be of major clinical importance, studies in cultured cells and animal models have shown that CCL3 inhibits osteoblast function, and that this influence is mediated by CCR1. However, as *Ccr1*-deficient mice display a severe impairment in osteoblastogenesis, instead of increased bone formation, it still remains to established, if and how a CCL3/CCR1 interaction influences physiological bone remodeling. Regardless of these open questions, it is quite important that there is also clinical relevance for an impact of CCL3 in human bone pathologies.

### CCL5

**Figure 2:**
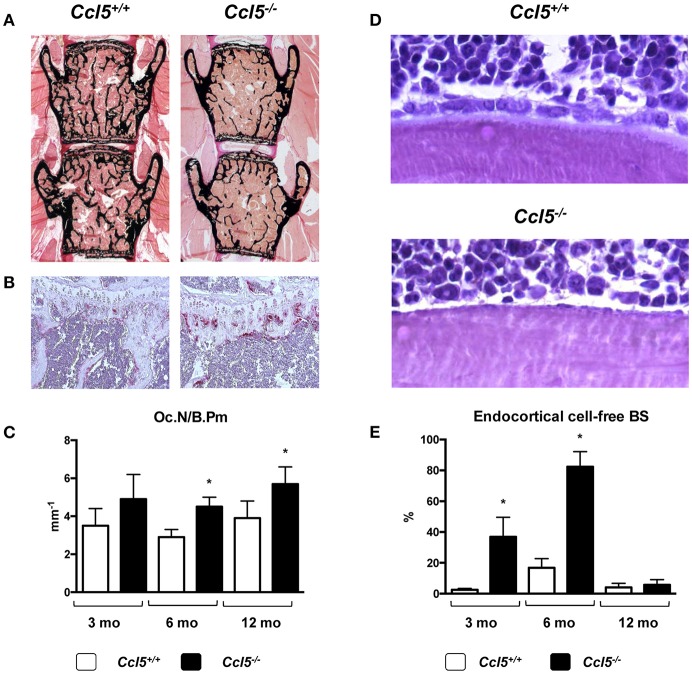
Bone remodeling phenotype of *Ccl5*-deficient mice. **(A)** Representative images of undecalcified spine sections (von Kossa/van Gieson-staining, mineralized bone appears black) from 6-month-old littermate mice with the indicated genotypes showing reduced trabecular bone mass in *Ccl5*-deficient animals. **(B)** Representative images of tibia sections stained for activity of the osteoclast marker TRAP (tartrate-resistant acid phosphatase, red staining) from the same mice demonstrating increased osteoclastogenesis in *Ccl5*-deficient animals. **(C)** Histomorphometric quantification of the osteoclast number per bone perimeter (Oc.N/B.Pm) in wildtype and *Ccl5*-deficient littermate mice at the ages of 3, 6, and 12 months. Asterisks indicate significant differences (*p < 0.05). **(D)** Representative images of undecalcified tibia sections (toluidine blue staining) from 6-month-old littermate mice with the indicated genotypes show that the majority of endocortical bone surfaces in *Ccl5*-deficient animals are not covered by osteoblasts. **(E)** Quantification of the endocortical osteoblastic cell-free bone surface (BS) in wildtype and *Ccl5*-deficient littermate mice does not only demonstrate the severity of this phenotype at 3 and 6 months of age, but also that this pathology is normalized in 12-month-old animals. Asterisks indicate significant differences (*p < 0.05). These data are based on a published study (57).

CCL5 (also known as RANTES) can bind to different receptors (CCR1, CCR3-5). All of them were found expressed in primary osteoblasts, and it was demonstrated that CCL5 acts as a chemoattractant for osteoblasts *in vitro* (97). Based on an unbiased screening approach, where we identified CCL5 and CCL2 as transcriptionally regulated genes after short-term administration of Wnt5a (98), we analyzed the skeletal phenotype of both, *Ccl2*^−/−^ and *Ccl5*^−/−^ mice (57). Whereas *Ccl2*^−/−^ mice, in line with previous findings by others (55, 56) displayed an increased trabecular bone mass with reduced numbers of osteoclasts, the bone remodeling phenotype of *Ccl5*^−/−^ mice was remarkably different. More specifically, 6-month-old *Ccl5*^−/−^ mice displayed osteopenia with increased osteoclast numbers, i.e., the opposite phenotype as observed in age-matched *Ccl2*^−/−^ mice. Moreover, more than 80% of the endocortical bone surfaces in 6-month-old *Ccl5*^−/−^ mice were not covered by either osteoblasts or bone-lining cells. Of note, this pathology was associated with an absence of F4/80^+^ osteal macrophages, which were previously shown to promote osteoblast formation at endocortical bone surfaces (99). Although these data indicated that CCL5 plays a critical role in the recruitment of osteoblast progenitor cells, it is important to state that this phenotype was only transiently observed, as it diminished with age (Figure 2).

In our opinion, this comparative study is potentially relevant in several regards. First, it shows that the deficiency of individual chemokines can cause entirely different skeletal phenotypes, thereby demonstrating the specificity of chemokine functions. Second, it underscores the importance of analyzing different skeletal elements and areas, since the phenotype of 6-month-old *Ccl5*^−/−^ mice was much more pronounced in the cortical bone compartment of tibia sections than it was in the trabecular bone compartment of spine sections. Third, and most importantly, the transient nature of the *Ccl5*^−/−^ phenotype, which is potentially explained by functional redundancy, raises the important question, if similar compensatory mechanisms exist in other mouse models and/or patients. If so, it might be required to study skeletal phenotypes of mouse models lacking specific chemokines or chemokine receptors at various ages and to identify, if possible, other chemokines with the ability to compensate a single gene deficiency. On the other hand, it is essentially not too surprising that inactivation of one specific chemokine does not translate into a severe and persistent bone pathology, which might also explain, why there is still no evidence for mutations in a chemokine-encoding gene as a cause of a monogenic skeletal disorder.

### CCL20

CCL20 (also known as MIP-3α), attracts T cells, B cells and dendritic cells via CCR6 and is important in the mucosal immune system. *In vitro* studies suggested a role for CCL20 in osteoclastogenesis. Here it was found that, upon stimulation with CCL20, primary human osteoblasts expressed elevated IL-6 levels (100). Likewise, treatment of human peripheral blood monocytes with conditioned medium from CCL20-treated osteoblasts induced osteoclast formation, which could be inhibited by neutralizing anti-IL-6 antibody. Thus, CCL20 indirectly affects osteoclastogenesis by inducing IL-6 expression *in vitro*. On the other hand, mice deficient for *Ccr6*, which encodes the sole receptor for CCL20, did not display a defect in osteoclast formation, indicating that this mechanism might not be relevant under physiological conditions (101). However, despite there was no phenotype related to osteoclastogenesis in either *Ccr6*^−/−^ mice or *Ccl20*^−/−^ mice, both models displayed reduced trabecular bone mass (101). This was attributed to decreased bone formation, as these mice had reduced osteoblast numbers. Moreover, the authors found that the expression of *Ccr6* and *Ccl20* increased in the course of osteoblast differentiation, that osteoblast differentiation *in vitro* was delayed in cells from *Ccr6*^−/−^ mice, and that CCL20 promoted the survival of wildtype osteoblasts. Thus, the CCL20/CCR6 axis seems to have a physiological role in the regulation of bone formation in mice, by regulating osteoblasts, but not osteoclasts.

On the other hand, studies of disease models and patients suggested, that CCL20 plays a role in pathological bone loss. For instance, breast cancer cells were shown to express CCL20 and this expression negatively correlated with survival in patients (102). In line with this, treatment of a breast cancer bone metastasis mouse model with a neutralizing anti-CCL20 antibody could inhibit metastasis and osteolysis (102). Furthermore, CCL20/CCR6 signaling was shown to play a role in MM. CCL20 expression in osteoblasts correlated with osteolytic lesions in MM patients, and MM cells were shown to induce osteoblastic CCL20 expression, leading to osteoclast recruitment (103). Besides in cancer, the CCL20/CCR20 axis was shown to be involved in inflammation-induced bone loss. Inflammatory mediators were shown to induce CCL20 expression in cultured osteoblasts and to stimulate the formation of pre-osteoclasts, while *in vivo* CCL20 was found to be induced in subchondral bone of RA patients (104).

While these data suggest a critical role of CCL20/CCR6 in pathological bone loss disorders, it is somehow surprising that the *Ccr6*-deficient mice only displayed reduced bone formation. Although this may raise critical questions about the suitability of mouse models for complex human pathologies, the comparative analysis of mice deficient in specific chemokines and their receptors is undoubtfully informative, especially since the discrepancy of the respective phenotypes clearly demonstrates that there is true specificity regarding chemokine influences on bone remodeling cell types.

### Additional CC-Chemokines With Putative Influence on Bone Remodeling

Besides the four CC-chemokines discussed above, there are additional studies providing evidence for other family members as regulators of bone remodeling cell types. Although their (patho)physiological impact needs to be further investigated, it is certainly relevant to refer to the respective studies in the present review article.

**CCL4** (also known as MIP1-β), which can bind to CCR1 and CCR5, was shown to be induced during osteoclast differentiation of RAW264.7 macrophages. Moreover, neutralization of CCL4 inhibited RANKL-induced osteoclast migration, but not their differentiation (105). In line with this observation, another study reported that treatment of mouse osteoclast progenitor cells with CCL4 did not influence RANKL-mediated osteoclastogenesis. However, the decrease in expression of its receptor CCR5 during osteoclast formation, was shown to be essential for osteoclastogenesis (106).

Finally, with respect to CC-chemokine receptors, there is evidence for a role **CCR3** in bone remodeling. CCR3, which binds several ligands, including CCL5 and CCL11, is highly expressed on eosinophils and basophils. Circulating human monocytes were also shown to express CCR3 and this expression was negatively correlated with bone mineral density (86). Therefore, the skeletal phenotype of mice deficient for *Ccr3* was evaluated (107). *Ccr3*^−/−^ mice showed increased bone mineral density, and the authors hypothesized that this was due to effects on both, osteoclasts and osteoblasts. However, the study did not clarify the underlying cellular mechanisms. In another study it was found that the pro-inflammatory chemokine **CCL11** (also known as eotaxin), which predominantly binds to CCR3, is elevated in plasma of osteoarthritis patients (108). CCL11 was further identified to be the most significantly induced chemokine in the early phases of RA (109). In a bone inflammation mouse model, CCL11 was shown to be expressed by osteoblasts, concomitant with increased osteoclastogenesis and bone resorption, and that treatment of osteoclasts with CCL11 increased their resorptive activity on bone slices (110).

Taken together, there is huge complexity of the chemokine system, where certain receptors bind different ligands, and where deficiency of specific chemokines is potentially compensated by others. On the other hand, there are distinct bone phenotypes reported for various mouse models, where the lack of one chemokine or its receptor causes cell-specific impairments. Together with the data obtained in these models and/or patients with inflammatory bone loss or metastatic bone disease, the collective findings provide strong evidence that at least some CC-chemokines and their receptors are relevant in bone remodeling regulation. The same applies for CXC-chemokines, which will be discussed in the next section.

## Influence of CXC-chemokines on Bone Remodeling in Health and Disease

Similar to the CC-chemokines there is also strong evidence for the impact of specific CXC-chemokines on skeletal cell types under physiological and pathological conditions. We will again focus on the most established and/or relevant ligands, i.e., CXCL2, CXCL9, and CXCL12 in the following paragraphs. Whereas, CXCL2/CXCR2 signaling has again been linked to osteoclastogenesis, CXCL9 may play a unique role in the coupling of angiogenesis and bone formation. Moreover, the probably best established chemokine receptor pair, CXCL12/CXCR4, plays a key role in recruiting specific cell types into the bone marrow microenvironment, which is particularly relevant in metastatic bone disease. Again, the impact of specific chemokine influences on either osteoclast or osteoblast differentiation are depicted in the simplified schematic representation (Figure 1).

### CXCL2

CXCL2 (also known as MIP2-α) recruits neutrophils during inflammation via its receptor CXCR2 and is mainly produced by monocytes and macrophages. CXCL2 was shown to stimulate osteoclast formation *in vitro*, and the same was reported for an alternative CXCR2 ligand i.e., CXCL1 (111). Of note, this finding was made in the context of a study analyzing the role of CXCR2 signaling in marrow adipocyte-driven osteoclastogenesis (111). More specifically, adipose bone marrow, which commonly occurs in aging and obesity, was shown to induce osteoclast formation by expressing increased levels of CXCL1 and CXCL2, which in turn could be inhibited by antagonizing CXCR2. A different study reported that osteoclast precursor cells also expressed CXCL2 upon RANKL-stimulation and that osteoclast formation could be blocked by antagonizing CXCR2 (112). *In vivo* studies could confirm the pro-osteoclastogenic function of CXCL2. In mice, the injection of CXCL2 induced calvarial osteolysis (112), while osteolysis after LPS treatment was attributed to increased CXCL2 expression, since the LPS effect was be blocked with a neutralizing anti-CXCL2 antibody (113). The potential human relevance of CXCL2 is supported by two studies. In fact, CXCL2 was found to be induced in bone tissue surrounding bacterially infected implants (114), and patients with RA had elevated CXCL2 levels in their synovial fluids and sera (112).

A very recent publication demonstrated that CXCL2 might also inhibit osteoblast differentiation (115). In fact, osteoblasts in ovariectomized mice were shown to express increased levels of CXCL2 compared to sham operated controls, while injection of a neutralizing anti-CXCL2 antibody into the femoral cavity of these mice alleviated osteoporosis. Additionally, *in vitro* experiments showed that overexpression of CXCL2 in osteoblasts increased their proliferation at the expense of differentiation by inhibition of ERK1/2 signaling upstream of RUNX2, a transcription factor required for osteoblastogenesis. On the other hand, mice deficient for CXCR2 were smaller and lighter compared to wildtype littermates, had a lower trabecular bone volume with reduced cortical BMD and thickness, and their long bones had decreased mechanical properties (116). Also, the healing of calvarial defects in *Cxcr2*^−/−^ mice was delayed. Surprisingly however, no differences in either number or activity of osteoblasts and osteoclasts were found in *Cxcr2*^−/−^ mice. The authors argued that the role of CXCR2 in bone was rather related to its pro-angiogenic function and less to its effect on skeletal or immune cells. The fact that CXCR2 binds various chemokines with different functions (CXCL1-3, CXCL5-8), and is expressed by a variety of cells, might explain why the analysis of *Cxcr2*^−/−^ mice provided contradicting results (43).

In conclusion, there is *in vitro* and *in vivo* evidence indicating that CXCL2 influences bone remodeling by promoting osteoclastogenesis and inhibiting osteoblast differentiation. Whether these effects are mainly mediated by CXCR2 remains to be established, and the same applies for the potential relevance of CXCL1/CXCR2 signaling for physiological and pathological bone remodeling in humans.

### CXCL9

CXCL9 (also known as MIG) is an ELR-negative, angiostatic chemokine which is strongly induced by interferon-ɤ (INF_ɤ_). Similar to CXCL10 and CXCL11, CXCL9 exerts its immunological function through CXCR3, which is found on T cells and endothelial cells (117, 118). The main immunological role of CXCL9 is to attract CD4^+^ Th1 cells and CD8^+^ effector T cells to sites of inflammation. A recent publication by Huang et al. (119) has suggested an additional role for CXCL9 in the regulation of bone remodeling and vascularization. It was shown that osteoblasts constitutively express CXCL9 to regulate bone angiogenesis and osteogenesis. More specifically, in order to study the role of mammalian target of rapamycin complex 1 (mTORC1) signaling in bone remodeling, the authors generated mice with either constitutively activated or inactivated mTORC1 in mature osteocalcin-expressing osteoblasts. The major factor influencing osteogenesis and angiogenesis, which was positively regulated by mTORC1, was identified as CXCL9. It was further shown that CXCL9 inhibited angiogenesis by sequestering VEGF and preventing its binding to VEGFR. Moreover, CXCL9 was shown to inhibit osteoblast proliferation, differentiation and mineralization *in vitro* through a VEGF-dependent mechanism.

Of note, our own work related to the skeletal phenotype of mice deficient for fetuin-A (also known as α2-HS glycoprotein, encoded by the *Ahsg* gene), further suggested a critical role for CXCL9 during endochondral ossification (120). Fetuin-A is a hepatic plasma protein with high affinity to calcium phosphate, which explains its high abundance in the mineralized bone matrix (11, 121–123). Fetuin-A has been established as an important inhibitor of ectopic calcification (124), and shortened femoral bones in *Ahsg*^−/−^ mice indicated a role for this protein in endochondral ossification (125, 126). We found that *Ahsg*^−/−^ mice develop epiphysiolysis in their distal femora, which prompted us to perform a transcriptome analysis of the growth plates prior to growth plate slippage (120). The by far most strongly induced gene in *Ahsg*^−/−^ growth plates was *Cxcl9* with an increase of >500-fold compared to wildtype littermates. In line with the findings by Huang et al. (119), we additionally identified a decreased number of capillary loops at the chondro-osseous junction in *Ahsg*^−/−^ mice. These data suggest that excessive CXCL9 production in the growth plate of *Ahsg*^−/−^ mice causes their epiphysiolysis phenotype, yet there are further experiments needed to demonstrate such causality.

In our opinion, the combined findings regarding CXCL9 expression in skeletal cell types, are potentially relevant, since recent studies have shown that vascularization not only serves the purpose of blood supply, but also fulfills very specific developmental and functional roles (127, 128). It was shown that a specific subset of bone sinusoidal endothelial cells, which are characterized by high expression of endomucin and CD31, actively promote osteogenesis and in this manner couple vascularization and bone formation (129, 130). As chemokines, in particular CXC-chemokines, regulate inflammation, bone remodeling as well as angiogenesis it would be highly interesting to study them in the context of endochondral ossification. In this regard, CXCL9 is a good candidate molecule, yet the skeletal phenotype of a corresponding mouse deficiency model has not been analyzed to date.

### CXCL12

CXCL12 (also known as SDF-1) and its receptor CXCR4 represent one of the best studied chemokine/receptor pairs in several regards. The CXCL12/CXCR4 axis is crucial during development, as demonstrated by the fact that mice deficient for *Cxcl12* or *Cxcr4* die prenatally due to various defects in cardiac and brain development (131–133). Furthermore, CXCL12 is pro-angiogenic (despite being ELR-negative) and recruits CXCR4-expressing endothelial progenitors (134, 135). The CXCL12/CXCR4 axis is known as the most important pathway regulating the homing of HSC and developing innate immune cells into the bone marrow niche (136). In this manner, a pool of HSC is retained in the adult bone marrow niche, and adult mice with an induced deletion of *Cxcr4* have severely reduced numbers of bone marrow HSCs (136). Two back-to-back publications highlight the importance of osteoblasts and their progenitor cells in forming specific niches for HSC by specifically deleting *Cxcl12* in different cells of the bone marrow niche, including MSCs, osteoprogenitors or mature osteoblasts (44, 45). By expressing CXCL12, perivascular, endothelial and skeletal progenitor cells are crucial to maintain and support distinct subsets of hematopoietic progenitors in the bone marrow (137, 138). Bone marrow stromal cells, which can differentiate into osteoblasts, chondrocytes, adipocytes, and other different cell types, were shown to express CXCL12 and CXCR4, yet the expression of CXCL12 decreased with increased osteogenic differentiation (139). Of note, there is one cell type which expresses CXCL12 at very high levels, which is termed CXCL12-abundant reticular (CAR) cell. More specifically, CAR cells reside in the bone marrow niche surrounding sinusoidal endothelial cells, as well as in the endosteal niche. They are considered to be the major source of CXCL12 in the bone marrow (136). Furthermore, a specific subset of CXCR4^+^CD45^−^ pluripotent MSCs was identified in mouse bone marrow, which expresses high levels of CXCL12, but low levels of RANK and RANKL (140). The authors proposed that these cells represent a specific microenvironment, which supports osteoclastogenesis while not being directly involved in the RANKL signaling axis.

Apart from its roles in development, angiogenesis and stem cell homing, there is evidence from *in vitro* and *in vivo* studies that CXCL12 directly interacts with skeletal cells to regulate bone remodeling. RAW264.7 macrophages were shown to express CXCR4, and this expression decreased during RANKL-mediated osteoclastogenesis (141). Furthermore, CXCL12 acts as a chemoattractant for RAW264.7 cells, enhancing their migration through collagen, and increasing their MMP9 expression. An increased expression of MMP9 as well as an increased resorption of calcium phosphate chips was reported for human osteoclasts, which were differentiated in the presence of CXCL12 (142). CXCL12 was also shown to increase bone resorption in cultured human primary osteoclasts and induce resorption-related gene expression (*Ctsk, Mmp9*, and *Trap*), while this effect could be inhibited by the CXCR4-selective antagonist T140 (143).

The CXCL12/CXCR4 axis also plays important roles during bone loss induced by metastasis and MM. First of all, the CXCL12/CXCR4 interaction is critical for the recruitment of metastatic cancer cells into the bone marrow niche, since these cells, by expressing CXCR4, essentially hijack the homing mechanism for hematopoietic cells (144, 145). Furthermore, one study showed that MM patients had elevated plasma levels of CXCL12 which correlated with the occurrence of osteolytic bone lesions, and MM cells were shown to express significant amounts of CXCL12 (143). Interestingly, the CXCR4-specific inhibitor T140 reduced *in vitro* osteoclast formation which was stimulated by conditioned medium from an MM cell line, which contained high levels of CXCL12. Another study from the same group demonstrated a positive correlation between plasma levels of CXCL12 in MM patients and the bone resorption marker CrossLaps (146). It was further shown that intratibial injection of MM cell lines into mice induced focal osteolytic lesions proximal to the tumor, which could be reduced by T140, while osteolysis was increased when the tumor cells overexpressed CXCL12 (146). Taken together, by expressing CXCL12, MM cells recruit osteoclast precursors to the bone, thereby inducing osteolysis. Moreover, an involvement of CXCL12 in both RA and osteoarthritis has been demonstrated in numerous studies, where it affects synovial fibroblasts, immune cells and endothelial cells, and promotes the loss of bone and cartilage (147). The CXCL12/CXCR4 axis is therefore a promising drug target in RA, and treatment of mice with collagen-induced arthritis with the CXCR4-specific antagonist AMD3100 was shown to reduce disease severity (148).

Several studies demonstrated that the CXCL12/CXCR4 signaling pathway is not only involved in osteoclast formation, but also in osteoblast differentiation. It was shown that CXCR4 regulates osteoblast differentiation in cooperation with BMP signaling, and that mice with a conditional deletion of *Cxcr4* in osterix-expressing cells were osteopenic due to a defect in osteoblastogenesis (149). Moreover, primary osteoblasts from these mice were less responsive to treatment with BMP2 or BMP6, suggesting a coupling between BMP-signaling and the CXCL12/CXCR4 axis. In a subsequent study, it was shown that the expression of CXCR4 and CXCL12 in bone marrow-derived MSCs decreases with age, concomitant with decreased potential for *in vitro* osteogenic differentiation in response to BMP2 stimulation or osteogenic medium (150). Here the restoration of CXCR4 expression in bone marrow cells of old mice corrected their osteogenic differentiation defect. It was furthermore demonstrated that CXCL12 enhanced osteogenic differentiation of stromal cells which were transduced to express higher levels of CXCL12 (139). In line with this, mice with a deletion of CXCR4 in mature *Col1a1*-expressing osteoblasts were shown to have a decreased bone mass and decreased bone formation (151). Furthermore, a recent study showed that the deletion of *Cxcl12* in *Prx1*-expressing limb mesenchyme or osterix-expressing osteoblast progenitors, but not in mature osteoblasts, induced marrow adiposity and reduced trabecular bone volume (152). Thus, deletion of *Cxcl1*2 in osteoblast progenitor cells or early osteoblasts increased their adipogenic differentiation at the expense of osteogenic differentiation. Furthermore, expression of osteogenic markers, parameters of bone formation and osteoblast numbers were reduced in mice with a deletion of *Cxcl12* in *Prx1*-expressing cells, while osteoclast formation and activity were not affected. In contrast, deletion of *Cxcr4* in *Prx1*-expressing cells similarly led to a reduction in bone formation, but it did not increase marrow adiposity (152). Thus, limb mesenchymal cells regulate osteogenesis in a cell-autonomous manner through CXCL12, while the modulation of adipocyte differentiation occurs through other mechanisms.

In line with these findings, CXCL12 has been shown to regulate fracture healing through BMP2 signaling (153). More specifically, BMP2 signaling controlled the spatial and temporal expression pattern of CXCL12 by BMP2^+^ CXCL12^+^ perivascular endosteal cells, which were recruited to the fracture site. Deficiency of *Bmp2* in mice led to an induction of *Cxcl12* expression, leading to a deranged angiogenic response during fracture healing, which could be corrected by treatment with AMD3100 (154). Furthermore, the role of CXCL12/CXCR4 signaling in bone healing was studied in a mouse femoral bone fracture model (155). Here, *Cxcl12* mRNA expression was shown to increase during fracture healing, especially in the periosteal region. Treatment with a CXCL12-neutralizing antibody or the antagonist TF14016, a more stable analog of T140, inhibited the formation of new bone (156). The study also showed that CXCL12 recruited MSCs for bone formation during fracture repair and was also important for vascularization during bone fracture healing. Another study showed that when *Cxcl12* was deleted in *Tie2*-expressing endothelial progenitor cells, the fracture callus was less vascularized and fracture healing was delayed (157).

Finally, the CXCL12/CXCR4 axis was shown to be involved in endochondral ossification. One study in E18.5 mice showed that CXCR4 was expressed by proliferative chondrocytes, while CXCL12 was expressed by prehypertrophic and hypertrophic chondrocytes in the growth plate (149). Conditional deletion of *Cxcr4* in osterix-expressing cells, which resulted in a 70% reduction in CXCR4-positive growth plate chondrocytes, led to a disorganization of the growth plate and a decrease in growth plate proliferation. Another publication showed that in newborn mice, CXCR4 was predominantly expressed by hypertrophic chondrocytes, while CXCL12 was expressed in the adjacent bone marrow (158). Here it was shown that CXCR4/CXCL12 signaling induced chondrocyte hypertrophy and that this was regulated in a positive feedback-loop, which was mediated by RUNX2.

**Figure 3:**
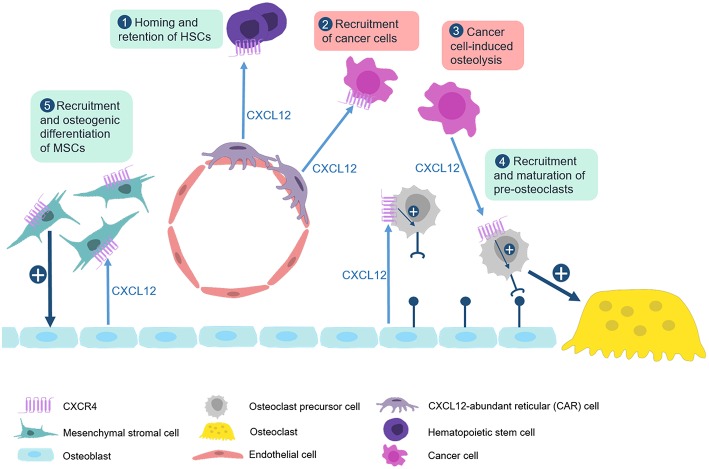
The CXCL12/CXCR4 axis in physiological and pathological bone remodeling. Numerous studies have established that the CXCL12/CXCR4 axis is not only required for homing of hematopoietic stem cells, but also for the regulation of bone remodeling cell types in physiological and pathological conditions. (1) CXCL12, which is predominantly expressed by CXCL12-abundant reticular (CAR) cells, binds to CXCR4 on hematopoietic stem cells to recruit them to bone microenvironment. (2) This mechanism is also used by CXCR4-expressing metastatic cancer cells which explains their recruitment to the bone marrow niche. (3) CXCL12 expression by multiple myeloma cells enhances recruitment and maturation of pre-osteoclasts by inducing RANK expression. (4) Osteoblasts also express CXCL12 to physiologically regulate migration and maturation of osteoclast progenitor cells. (5) CXCL12 additionally cooperates with BMP signaling to promote osteogenic differentiation of mesenchymal stromal cells.

Taken together, there is a huge amount of evidence, both *in vitro* and *in vivo*, showing that CXCL12 has remarkable influences in several aspects of skeletal biology (Figure 3). Through interaction with CXCR4 it promotes osteoclastogenesis, but it also induces osteogenic differentiation of mesenchymal stromal cells in cooperation with BMP signaling. The CXCL12/CXCR4 axis additionally regulates growth plate chondrocyte proliferation and hypertrophy during development, at least in mice. The most critical impact however is probably related to cancer metastases, since the respective tumor cells apparently hijack the CXCL12-mediated homing to the bone marrow by expressing CXCR4. In this regard, blockade of CXCR4 might be a valuable approach to prevent the detrimental interaction of cancer and bone remodeling cells and the development of osteolytic lesions. Currently, the most established CXCR4 antagonist is AMD3100 (Plerixafor) (154, 159–161). Originally developed as an antiviral agent against the replication of HIV, this drug is now widely used for the mobilization of HSC for autologous stem cell transplantation in lymphoma and MM patients. However, the low oral bioavailability of Plerixafor makes it less suitable for longer treatments. Therefore, the safety and efficacy of other CXCR4 antagonists is currently being evaluated in clinical trials (162, 163).

### Additional CXC-Chemokines With Putative Influence on Bone Remodeling

In addition to the three CXC-chemokines discussed above, it is again important to refer to studies on the putative impact of other CXC-chemokines as regulators of skeletal remodeling. In these cases the *in vivo* significance is less established so far, which however does not mean that the influences of the respective molecules on skeletal cell types are less relevant.

**CXCL8** (also known as IL-8) is a ligand for both, CXCR2 and CXCR1. Similar to CXCL2, it is secreted by macrophages and also by epithelial and endothelial cells. Its role in bone remodeling has mainly been studied *in vitro*. First, osteoblasts and osteoclasts were shown to express CXCL8 upon stimulation with inflammatory mediators (164, 165). Primary human osteoblasts stimulated with CXCL8 expressed elevated IL-6 levels and conditioned medium from these cells induced osteoclast formation in human peripheral blood monocytes, which could be inhibited by neutralizing anti-IL-6 antibody (100). Furthermore, treatment of human osteoclast precursor cells with CXCL8 in the presence of M-CSF was shown to induce the formation of TRAP^+^ osteoclasts, and it was found that these cells were able to resorb bone in the absence of RANKL (166). Thus, CXCL8 stimulates bone resorption through direct and indirect mechanisms. A role for CXCL8 in bone metastatic disease was demonstrated in studies with breast cancer cells (166, 167). More specifically, the bone-tropic subclone MDA-MET derived from the human breast cancer cell-line MDA-MB-231 was found to secrete high levels of CXCL8. After tibial injection of MDA-MET, all recipient mice developed large osteolytic bone metastases, whereas treatment with a CXCL8-neutralizing monoclonal antibody prevented tumor formation in 85% of the mice (167). Finally, breast cancer patients with bone metastases were shown to have elevated CXCL8 plasma levels compared to patients without metastasis, and the CXCL8 plasma levels correlated with increased bone resorption (167). These data suggested that CXCL8 could be a promising drug target for breast cancer bone metastasis.

Like CXCL2 and CXCL8, **CXCL5** (also known as LIX) is a chemoattractant for neutrophils via the receptor CXCR2. *In vitro*, CXCL5 was found to be induced by IL-17 in osteoblasts (168). *In vivo*, increased CXCL5 expression was found in individuals with Paget's disease of bone (169), where a local dysregulation of bone remodeling causes high bone turnover (170). More specifically, these patients displayed a 180-fold higher expression of *CXCL5* in bone marrow cells, and a 5-fold increase of CXCL5 serum levels (169). By utilizing chromatin immunoprecipitation, the authors additionally found that CXCL5 increased RANKL expression in human bone marrow-derived stromal cells through the phosphorylation of CREB.

Finally, **CXCL10** (also known as IP-10), similar to CXCL9, also binds to CXCR3. A potential role for CXCL10 in bone remodeling was identified in mice with an osteoblast-specific deletion of menin-1 (171), which develop an osteoporotic phenotype due to increased bone resorption. In an unbiased approach, it was shown that osteocytes from these mice express increased levels of CXCL10, and that treatment with anti-CXCL10 antibody could normalize osteoclast activity *in vivo*. In addition, it was reported that CXCL10 is involved in the recruitment of CXCR3-expressing cancer cells to the bone marrow leading to bone metastasis formation, induction of osteoclast differentiation and osteolysis, while treatment with anti-CXCL10 antibody decreased metastasis formation *in vivo* (172). Finally, CXCL10 has been shown to promote bone loss in a mouse model of collagen-induced arthritis (173).

Again, similar to the CC-chemokines, these latter examples illustrate that there are many different studies supporting a critical function of specific chemokines in physiological and pathological bone remodeling, most of them performed in cultured cells or in mouse deficiency models. The large amount of significant influences reported by many different investigators raises the critical question about the relative importance of the respective findings. Although it is evident that some ligand receptor pairs are better studied than others, it still remains to be established, which of these interactions are truly relevant for (patho)physiological skeletal remodeling regulation in humans. On the other hand, the same level of complexity applies for other key players in osteoimmunology, i.e., cytokines. In that case, it was indeed important that cumulative evidence was obtained in different groups of patients, thereby demonstrating, for instance, that IL17A does not only increase osteoclastogenesis in cell culture assays or mice, but also in specific patient groups (27–31). Based on these arguments, there is probably even more research necessary to clearly define chemokine receptor pairs, which could also serve as drug targets for patient treatment.

## CX3CL1

In addition to CC- and CXC-chemokines, there is one chemokine with pronounced influence on bone remodeling, i.e., CX3CL1, which does not fall into the two classical categories. Of note, CX3CL1 (also known as fractalkine) is a membrane-bound chemokine, which can be proteolytically processed to release a soluble domain that attracts cells expressing the receptor CX3CR1. Moreover, the uncleaved membrane protein can mediate a direct cell contact between *Cx3cl1*- and *Cx3cr1*-expressing cells. It was shown that CX3CL1 is expressed by osteoblasts, while its receptor CX3CR1 is present on osteoclast progenitors (174). Whereas, the soluble domain of CX3CL1 induces chemotaxis of osteoclast progenitors, the interaction of membrane-bound CX3CL1 expressed by osteoblasts with CX3CR1 on osteoclast progenitors was found to induce terminal differentiation of the latter. Moreover, administration of a CX3CR1-neutralizing antibody inhibited not only the osteoclastogenesis-promoting influence of co-cultured osteoblasts, but also the number and activity of osteoclasts in wildtype mice (174).

The physiological relevance of these findings was supported by skeletal phenotyping of CX3CR1-deficient mice, which display moderately, yet significantly increased trabecular bone mass, mostly explained by reduced numbers of osteoclasts (175). *Ex vivo* experiments with primary CX3CR1-deficient osteoblasts and/or osteoclasts suggested that this phenotype can be explained by a dual mechanism, i.e. a reduced RANKL/OPG ratio produced by CX3CR1-deficient osteoblasts, and a cell-autonomous osteoclastogenesis defect of CX3CR1-deficient bone marrow cells. Another *in vivo* study of irradiation-induced osteoclastogenesis in mice showed, that circulating pre-osteoclasts, displaying high expression of CX3CR1, are attracted by vascular expression of CX3CL1 (176). More specifically, bone loss in these mice was less pronounced, when the transplanted bone marrow cells were derived from CX3CL1-deficient mice or when a CX3CR1-neutralizing antibody was injected. In line with these findings, the expression of CX3CL1 in synovial fibroblasts has further been linked to osteoclast-mediated bone destruction (177). Moreover, CX3CL1 expression in osteoblasts was found remarkably induced by inflammatory cytokines, and CX3CR1 was identified as a marker for inflammatory osteoclasts (178–180).

Overall, these data suggest that CX3CL1 promotes osteoclast-mediated bone loss. Importantly, a neutralizing antibody against CX3CL1 is already studied in clinical trials for the treatment of inflammatory disorders, including RA (177). So far it has been shown that this monoclonal antibody (E6011) is safe and well-tolerated in RA patients, yet its efficacy for reducing joint destruction remains to be studied in larger cohorts (181).

## Atypical Chemokine Receptors

As stated in the introduction, the complexity of chemokine signaling is further enhanced by the existence of four atypical chemokine receptors (ACKR1-ACKR4), which do not induce classical G-protein coupled signaling (40–42). While ACKR1 primarily acts by transporting the bound chemokine across the cell (182), ACKR2, ACKR3, and ACKR4 have been identified as scavenging receptors, which induce the degradation of the sequestered chemokine (42). Furthermore, ACKR2 and other scavenging ACKRs regulate the relocalization of β-arrestin from the cytoplasm to the cell surface (42), which in turn controls the activity and internalization of G-protein coupled receptors. Although there are only few studies so far, which evaluated the potential role of atypical chemokine receptors in bone remodeling, it is relevant to discuss these data, since ACKRs are now considered as key regulators of chemokine signaling.

As stated above, **ACKR1** (also known as the human blood group antigen Duffy antigen receptor for chemokines, DARC) does not induce ligand degradation, unlike ACKR2-4. Instead, after binding of the ligand, ACKR1 is internalized and transports the chemokine across the cell, a process known as transcytosis (182). This occurs for instance on endothelial cells, where ACKR1 transports chemokines across the endothelial cell barrier in order to regulate leukocyte transmigration (183). Since ACKR1 was identified as a quantitative trait locus for bone mineral density in mice, the skeletal phenotype of *Ackr1*-deficient mice was studied (184). These mice displayed a higher bone mineral density compared to wildtype controls possibly explained by reduced osteoclastogenesis. This conclusion was supported by the finding that an anti-ACKR1 antibody blocked the formation of osteoclasts *in vitro*. Moreover, when LPS was injected above the calvaria, *Ackr1*-deficient mice showed a decrease in monocyte recruitment and of TRAP-positive osteoclasts at the injection site compared to wildtype controls (185). Given the known biological function of ACKR1, this decoy receptor might be involved in the transcytosis of pro-inflammatory chemokines through the endothelial cell barrier and in this manner regulate osteoclast recruitment.

The scavenger receptor **ACKR2** (also known as D6), is internalized into the endosome and is transported back to the cell surface independent of ligand binding (186). When a chemokine is bound to ACKR2, it will detach inside of the endosome and is subjected to lysosomal degradation. As ACKR2 binds mostly pro-inflammatory chemokines, it functions to resolve chemokine-driven inflammation (187). One study investigated the role of ACKR2 during orthodontic tooth movement (OTM) (188). It was shown that ACKR2 was expressed during OTM in mature osteoclasts and early osteoblasts from wildtype mice. In *Ackr2*-deficient mice, osteoclast numbers, the expression of bone resorption markers and OTM were significantly increased. These findings are in principal agreement with the known biological function of ACKR2 as a scavenging receptor, and they suggest that therapeutic strategies increasing ACKR2 production might be useful to inhibit bone loss during inflammatory conditions.

**ACKR3** (also known as CXCR7) specifically binds CXCL12 and CXCL11 and can thus be regarded as a decoy receptor antagonizing the CXCR12/CXCR4 axis. As described above, mice deficient for *Cxcl12* or its receptor *Cxcr4* die prenatally due to various defects (131–133). Similarly, the majority of *Ackr3*-deficient mice died in the early postnatal phase due to cardiovascular defects, yet about 30% of these mice survived until adulthood (189). In reporter mice, ACKR3 was shown to be highly expressed in vascular endothelial cells, cardiomyocytes and also in osteocytes. Therefore, the skeletal phenotype was investigated at birth and at four weeks of age, however no differences between *Ackr3*-deficient mice and wildtype littermates were identified by μCT analysis. Moreover, no major differences were found after subjecting female mice to ovariectomy or male mice to orchidectomy. Thus, although ACKR3 was found highly expressed in osteocytes, it remains to be established, for instance by generating mice with cell-specific *Ackr3* deficiency, if this is linked to a functional role in bone remodeling.

Taken together, there is only a limited number of publications so far that addressed the influence of atypical chemokine receptors on physiological and pathological bone remodeling. Since ACKR2 mostly binds to proinflammatory chemokines, which were found to mediate a pro-osteoclastogenic influence, the respective findings can be regarded as the most promising ones. From a therapeutic perspective however, it would be advantageous to target a more specific interaction, as it is mediated by ACKR3.

## Concluding Remarks

As summarized in this review article, there is a huge amount of literature demonstrating that several chemokines and their respective receptors impact skeletal remodeling under physiological and pathological conditions. While the relevance of some influences needs to be supported by additional evidence, there are specific ligand-receptor pairs, which are truly established as regulators of bone remodeling cell types, based on the combined efforts by various investigators (Table 1). Despite the huge complexity of the chemokine system and probable functional redundancy, it is quite remarkable that many mouse models lacking specific ligands or receptors display a distinct impairment of their bone remodeling status. On the other hand, there is so far no evidence for mutations in specific genes encoding either chemokines or their receptors that would cause a monogenic skeletal remodeling disorder. Therefore, it is reasonable to speculate that chemokine signaling rather affects human bone remodeling in specific situations associated with either inflammation or the presence of tumor cells in the bone microenvironment. Since such diseases are highly prevalent, the accumulated knowledge summarized here could provide novel treatment options, by targeting chemokine signaling, for a large number of affected individuals. Based on these arguments it is still required to expand this research area in order to identify the most critical chemokine receptor pairs playing a role in human (patho)physiology.

**Table 1:** Influences of the most established chemokines on physiological and pathological bone remodeling.

**Ligand**	**Receptor**	**Impact on physiological bone remodeling**	**Impact on pathological bone remodeling**
CCL2/MCP-1	CCR2	Stimulation of osteoclastogenesis (55–57)	Fracture healing (74, 75) Osteoporosis (55, 58) PTH treatment (59–61) Bone metastasis (62–66) Multiple myeloma (67) Bacterial inflammation (69–71)
CCL3/MIP1-α	CCR1, CCR5	Stimulation of osteoclastogenesis (77–84)	Fracture healing (85) Osteoporosis (87) Multiple myeloma (77, 83, 91–93) Rheumatoid arthritis (88, 89) Bacterial inflammation (114) Osteoarthritis (108)
CCL5/RANTES	CCR4, CCR5, CCR1	Osteoblast migration and bone formation (57, 97) Inhibition of osteoclastogenesis (57)	
CCL11/Eotaxin-1	CCR3	Stimulation of osteoclastogenesis and bone formation (107)	Rheumatoid arthritis (109, 110) Osteoarthritis (108)
CCL20/MIP3-α	CCR6	Stimulation of osteoclastogenesis (100, 101) Osteoblast differentiation (101)	Bone metastasis (102) Multiple myeloma (103) Rheumatoid arthritis (104)
CXCL2/MIP2-α	CXCR2	Stimulation of osteoclastogenesis (111, 112, 116)	Bacterial inflammation (113, 114) Rheumatoid arthritis (112)
CXCL5/LINX	CXCR2		Paget's disease (169) Neutrophil recruitment (168)
CXCL8/IL-8	CXCR1, CXCR2	Stimulation of osteoclastogenesis (100, 166)	Bone metastasis (166, 167)
CXCL9/MIG	CXCR3	Inhibition of osteoblast differentiation (119) Inhibition of bone angiogenesis (119) Endochondral ossification (120)	
CXCL10/IP-10	CXCR3		Osteoporosis (144) Bone metastasis (145) Rheumatoid arthritis (173)
CXCL12/SDF-1	CXCR4	Stimulation of osteoclastogenesis (141–143) Stimulation of osteoblastogenesis (139, 149, 150, 152) Endochondral ossification (149)	Fracture healing (153, 155, 157) Bone metastasis (144, 145) Multiple myeloma (143, 146) Rheumatoid arthritis (142, 147, 148)
CX3CL1/fractalkine	CX3CR1	Stimulation of osteoclastogenesis (174–176)	Rheumatoid arthritis (177–180)

## Author Contributions

All authors listed have made a substantial, direct and intellectual contribution to the work, and approved it for publication.

### Conflict of Interest Statement

The authors declare that the research was conducted in the absence of any commercial or financial relationships that could be construed as a potential conflict of interest.
